# Direct solvent free synthesis of bare α-NiS, β-NiS and α-β-NiS composite as excellent electrocatalysts: Effect of self-capping on supercapacitance and overall water splitting activity

**DOI:** 10.1038/s41598-020-59714-9

**Published:** 2020-02-24

**Authors:** Ginena Bildard Shombe, Malik Dilshad Khan, Camila Zequine, Chen Zhao, Ram K. Gupta, Neerish Revaprasadu

**Affiliations:** 1 Department of Chemistry, University of Zululand, Private Bag X1001, KwaDlangezwa, 3886 South Africa; 2 Department of Chemistry, Pittsburg State University, Pittsburg, KS 66762 USA

**Keywords:** Chemistry, Materials science

## Abstract

Nickel sulfide is regarded as a material with tremendous potential for energy storage and conversion applications. However, it exists in a variety of stable compositions and obtaining a pure phase is a challenge. This study demonstrates a potentially scalable, solvent free and phase selective synthesis of uncapped α-NiS, β-NiS and α-β-NiS composites using nickel alkyl (ethyl, octyl) xanthate precursors. Phase transformation and morphology were observed by powder-X-ray diffraction (p-XRD), transmission electron microscopy (TEM) and scanning electron microscopy (SEM). The comparative efficiency of the synthesized samples was investigated for energy storage and generation applications, in which superior performance was observed for the NiS synthesized from the short chain xanthate complex. A high specific capacitance of 1,940 F/g, 2,150 F/g and 2,250 F/g was observed at 2 mV/s for bare α-NiS, β-NiS and α-β-NiS composite respectively. At high current density of 1 A/g, α-NiS showed the highest capacitance of 1,287 F/g, with 100% of Coulombic efficiency and 79% of capacitance retention. In the case of the oxygen evolution reaction (OER), β-NiS showed an overpotential of 139 mV at a current density of 10 mA/cm^2^, with a Tafel slope of only 32 mV/dec, showing a fast and efficient process. It was observed that the increase in carbon chain of the synthesized self-capped nickel sulfide nanoparticles decreased the overall efficiency, both for energy storage and energy generation applications.

## Introduction

As a step towards the implementation of sustainable energy development strategies, research on the design of high-performance energy storage and conversion systems is gathering renewed momentum^1–3^. Sodium ion batteries (SIBs), lithium-ion batteries (LIBs), and supercapacitors (SCs) are examples of the most studied energy storage devices^4,5^. Energy conversion systems on the other hand, constitute a series of electrochemical reactions occurring in an electrolytic cell or in a hydrogen-oxygen fuel cell^3^. Hydrogen is a clean and sustainable energy carrier, currently regarded as the best alternative fuel of the future^6,7^. Its generation via the electrocatalytic splitting of water is a commonly investigated energy conversion technology^8^.

The performance of both energy storage devices and energy conversion systems is largely influenced by the type of electroactive material employed. Generally, for energy conversion systems the goal is to develop low cost, earth-abundant and efficient electrocatalysts that will replace Pt-, Ir- and Ru- based compounds^9^, while for energy storage devices, the goal is to develop advanced electrode materials that can deliver high energy and power densities^10^. Carbon-based materials^11^, conductive polymers^12^, transition metal oxides^13^, nitrides^14^, carbides^14^, phosphides^15^, and sulfides^5^ are among the materials investigated for both energy storage and generation applications. Owing to their low cost, high electrochemical activity as well as mechanical and thermal stability, transition metal sulfides have demonstrated promising potential. They possess fascinating properties such as excellent redox reversibility, conductivity, and capacitance^5,16^.

Nickel sulfides constitute a class of transition metal sulfides with unique optical, electrochemical and magnetic properties^17–21^. The group consists of multiple compounds with distinct phases and stoichiometries including NiS, Ni_3_S_2_, NiS_2_, Ni_3+x_S_2_, Ni_3_S_4_, Ni_6_S_5_, Ni_4_S_3+x_, Ni_7_S_6_, and Ni_9_S_8_^22^. This rich chemistry gives the family of nickel sulfides a vast array of properties and applications^17–21^. Following their easy availability, low cost, and good electron transport properties, recent studies on nickel sulfides have mainly focused on their suitability as electroactive materials for SIBs^19^, LIBs^19^, supercapacitors^20^ and electrocatalytic water splitting^8,21^. The most widely investigated phases include Ni_3_S_2_, Ni_3_S_4_, NiS_2_ and NiS^19–21,23^. A recent study by Bhosale *et al*., reported the efficiency of a sulfur deficient nickel disulfide phase, NiS_1.97_, for photoelectrochemical hydrogen generation activity^24^.

Stoichiometric NiS can be obtained as α-NiS (hexagonal, Nickeline phase), or β-NiS (rhombohedral Millerite phase) depending on the synthetic route and reaction parameters employed^25–27^. The millerite β-NiS phase exhibits metallic properties^28^, while the α-NiS phase shows metal to semiconductor as well as paramagnetic to antiferromagnetic transitions^29^. Studies conducted by Kullerud and Yund on the Ni-S system suggest that transitions between the α- and β-NiS phases can occur at 379 ± 3 °C^22^. Both α- and β-NiS have been studied for supercapacitance and water splitting applications, achieving specific capacitances as high as 1122 F g^−1^ ^23^, and good catalytic performance^25,26^. In pursuit of better cycling stability and overall electrochemical performance, much effort has been directed towards the formation of heterostructures of NiS and other active materials such as Ni_2_P^30^, graphine oxide^31^, and metal chalcogenides^32–34^.

Nickel sulfides have been prepared by several routes and nanostructured materials of different properties have been obtained^16^. The use of single source molecular precursors has produced high quality nanomaterials of various types over the years^35–37^. However, the synthesis of phase pure nickel sulfide nanomaterials from single molecular precursors are limited, mixed-phase systems are normally generated. For instance, Gervas *et al*. employed a solvothermal route for the synthesis of Ni_3_S_4_ and Ni_3_S_2_ nanoparticles from dithiocarbamate precursors in which a mixture of other phases of nickel sulfide were also obtained^38^. Ghezelbash *et al*., carried out the solventless thermolysis of nickel thiolate precursors which resulted in a mixed phase system of NiS and Ni_3_S_4_^39^. Employing nickel xanthate precursors in the solution based synthesis of nickel sulfide nanoparticles and/or thin films has also resulted in impure phases of nickel sulfide amongst other pure phases^40,41^. Similarly, the use of long chain organic surfactants is known to provide better control over the size and morphology of the synthesized materials^42^. However, the presence of a significant amount of ligand/carbonaceous residue on the surface of the materials originating from the ligands has been shown to diminish materials’ end application^43,44^.

The synthesis of nanomaterials via thermolytic, solvent free decomposition of single molecular precursors has been demonstrated as a simple, economical, self-capping and highly efficient route^45^. The method avoids the use of long chain capping ligands, and thus offers the possibility of synthesizing nanomaterials with abundant exposed active sites for enhanced electrocatalytic performance^43,44^. Various single source precursors such as metal thiolates, metal salts of dithiocarbamates, xanthates, thiocarboxylates and thiotetrazoles have been employed in the synthesis of a variety of nanostructured materials^45–48^. In this work, we have employed the solventless approach to synthesize nickel sulfide using nickel xanthate complexes as single source precursors. The use of xanthate precursors is advantageous as the by-products generated by the decomposition of xanthate complexes are highly volatile and can be removed easily. Furthermore, size and morphology control of the synthesized nanomaterials can be achieved by varying the chain length of the precursor. α-NiS, β-NiS and an α,β-NiS composite were synthesized from respective xanthate complexes, and their energy generation and energy storage behavior was investigated. The effect of precursor chain length on the electrochemical properties of NiS was also examined.

## Experimental

### Chemicals

Carbon disulfide (≥99.5%), 1-octanol (≥99%), potassium hydroxide (85.0%), chloroform (min 99%), nickel (II) acetate tetrahydrate, acetone and hexane were purchased from Merck while potassium ethyl xanthogente (96%) was purchased from Sigma-Aldrich. All chemicals were used as received.

### Instrumentation

Elemental analysis of the precursors was performed on a Perkin-Elmer automated model 2400 series II CHNS/O analyzer. FTIR analysis was carried out on a Bruker FT-IR tensor 27 spectrophotometer and the spectra recorded in the 200–4000 cm^−1^ range. Thermogravimetric analysis of the complexes was done using a Perkin Elmer Pyris 6 TGA from 30 to 600 °C at 10 °C min^−1^ under N_2_ gas flow. TEM and HRTEM analyses of the synthesized nanoparticles were carried out using a JEOL 1400 TEM and JEOL 2100 HRTEM respectively. Samples of NiS nanoparticles were prepared by placing a drop of the particles dilute solution on Formvar-coated grids (150 mesh) for TEM, and holey carbon grids for HRTEM. The samples were allowed to dry completely at room temperature, viewed at accelerating voltages of 120 kV and 200 kV for TEM and HRTEM respectively. Further processing of the acquired images was done using soft imaging systems iTEM software (TEM) and Gatan camera with Gatan software (HRTEM). XRD analysis was performed on a Bruker AXS D8 diffractometer which uses nickel-filtered Cu Kα radiation (λ = 1.5418 Å) at 40 kV, 40 mA. Scans were recorded in the high angle 2θ range of 10–80° at a scan speed of 0.2 sec/step and an increment of 0.01314. The surface morphology of the particles was analyzed by a Zeiss Ultra Plus FEG Scanning Electron Microscope (SEM) equipped with an Oxford detector EDX at 20 kV which uses Aztec software for elemental analysis. The surface composition and chemical states of the synthesized NiS were examined using a Kratos Axis Ultra DLD X-ray Photoelectron Spectrophotometer.

### Synthesis of precursors

#### Synthesis of *bis*(O-ethylxanthato) nickel (II); complex (1)

In a typical reaction, an aqueous solution of nickel acetate tetrahydrate (1.2443 g, 5.0 mmol, in 25 mL of distilled water) was added drop-wise to the respective solution of potassium ethyl xanthogenate (1.603 g, 10.0 mmol, in 25 mL of distilled water) and stirred for 1 hour. The precipitates formed were then collected, washed with distilled water, dried under vacuum and recrystallized from chloroform. Elemental analysis for C_6_H_10_O_2_S_4_Ni: Calc. C, 23.91%; H, 3.35%; S, 42.51%; Ni, 19.49%. Found: C, 23.81%; H, 3.2%; S, 42.17%; Ni, 20.1%.

#### Synthesis of bis(O-octylxanthato) nickel (II); complex (2)

Complex (2) was synthesized by a method previously reported^41^. Briefly, potassium hydroxide (1.788 g, 0.03187 mol) was dissolved in excess of 1-octanol (40 mL) while stirring. Upon complete dissolution of KOH, the solution was cooled to 0 °C followed by a drop-wise addition of carbon disulfide (1.9 mL, 0.03187 mol). After an hour, the formed yellow precipitates were filtered, washed with hexane, dried under vacuum and recrystallized from acetone. The preparation of the complex involved a drop-wise addition of an aqueous solution of nickel acetate tetrahydrate (1.189 g, 5.0 mmol, in 25 mL of water) to the ethanolic solution of the potassium octyl xanthogenate (2.05 g, 10.0 mmol) while stirring. The solution was stirred for 1 hour and the precipitate formed was then collected, washed with distilled water, dried under vacuum and recrystallized from chloroform. Elemental analysis for C_18_H_34_O_2_S_4_Ni: Calc. C, 46.06%; H, 7.30%; S, 27.32%; Ni, 12.50%. Found: C, 46.06%; H, 7.34%; S, 27.21%; Ni, 12.24%.

### Synthesis of nickel sulfide nanoparticles

A ceramic boat containing 0.3 g of the precursor was placed in the center of a quartz tube and inserted in the furnace. The sample was then heated to the required temperature (200, 300, 350 or 400 °C) for one hour under N_2_ flow. After the specified time, the furnace was cooled to room temperature naturally. The product, in the form of black powder, was collected and used for further analysis.

### Electrochemical studies

Electrochemical investigation of the synthesized materials was carried out using Gamry Potentiostat by three-electrode system, according to the protocols previously reported^36,37^. A paste of the synthesized sample (80 wt.%), polyvinylidene difluoride (PVDF, 10 wt.%), and acetylene black (10 wt.%) was prepared in N-methyl pyrrolidinone (NMP). This paste was applied to pre-cleaned and weighted nickel foam, and then dried under vacuum at 60 °C for 10 hours and used as a working electrode. Ni foam (EQ-bcnf-16m) from MTI Corporation, USA, was used for this study with 99.99% purity. The surface density was 346 g/m^2^. Conducting acetylene black (EQ-Lib-AB), from MTI corporation, USA, was used as commercial carbon having particle size of 35–40 nm. A platinum wire and saturated calomel electrode (SCE) were used as counter and reference electrodes, respectively. All the experiments for energy storage and electrocatalysis were carried out using 3 M and 1 M KOH electrolyte, respectively. Charge storage capacity was measured using cyclic voltammetry (CV) and galvanostatic charge-discharge (CD) at various scan rates and current densities, respectively. Electrocatalytic properties of the synthesized electrodes were studied using linear sweep voltammetry (LSV), cyclic voltammetry and chronoamperometry (CA). LSV was performed at a scan rate of 2 mV/s for both OER and HER measurements. Electrochemical impedance spectroscopic (EIS) was performed during all the tests in the frequency range of 0.05 Hz to 10 kHz with an applied AC amplitude of 10 mV.

## Results and Discussion

### Characterization of the precursors and nanoparticles

The synthesized precursors were characterized by CHN, IR spectroscopy and TGA (for the complexes only), and the results are comparable to those previously reported^41,49^. The complexes were thermally decomposed in an inert environment to produce nickel sulfide. The decomposition mechanism of the xanthate complexes is well known and is similar to that observed in the Chugaev elimination reaction^50^. The advantage of using xanthate complexes is that the by-products (COS and an alkene) generated during thermal decomposition are highly volatile and hence easily removed, leaving behind a pure metal sulfide. The generation of metal sulfides by the decomposition of xanthate complexes is feasible for scalable synthesis and the process is efficient in terms of cost as well as time.

Since the synthesis of nickel sulfide nanoparticles involves the thermal decomposition of the respective molecular precursors, thermal analysis of the two complexes was done to investigate their decomposition behavior. The resulting thermograms are shown in Figure S1, ESI. Both thermograms display a single step decomposition pattern which is attributed to the loss of the organic moiety leaving nickel sulfide as the final product. The thermogram of complex **(1)** displays a weight loss of about 72%, with an onset temperature of 155 °C. A complete decomposition was observed at 212 °C, leaving about 28% of the residue. The thermogram of complex **(2)** shows a relatively higher onset temperature of about 185 °C, and a weight loss of about 83%. A complete decomposition was observed at 285 °C with about 17% of the original material left behind. These results correlate with the theoretical values calculated for nickel (II) sulfide as the residue (29% and 19% for complexes (**1**) and (**2**) respectively). The results also reveal complex **(2)** to be more stable than complex **(1)** and this can be ascribed to the chain length of the precursor^51^.

**Figure 1:**
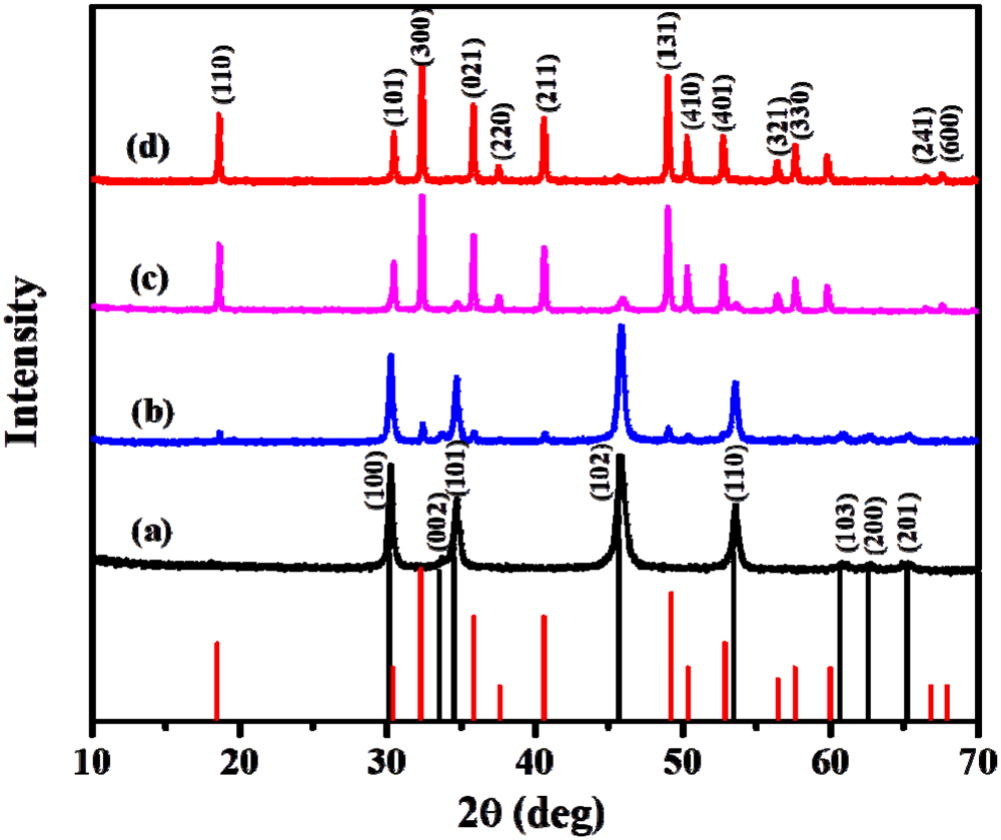
p-XRD patterns of NiS nanoparticles synthesized from complex (**1**) at (**a**) 200 °C (ICDD # 03-065-3419), (**b**) 300 °C, (**c**) 350 °C and (**d**) 400 °C (ICDD # 00-003-0760).

The p-XRD patterns of the particles synthesized from complex (**1**) in the temperature range of 200–400 °C are shown in Fig. 1. Both α- and β- polymorphs of NiS were synthesized depending on the reaction temperature. At 200 °C, a highly crystalline, pure hexagonal (α-NiS) phase was obtained (ICDD # 03-065-3419), (Fig. 1a). When pyrolysis of complex **(1)** was carried out at 300 °C, some additional, low intensity peaks, which were found to be the characteristic peaks of the β-NiS phase were formed (ICDD # 00-003-0760). A composite of the α-NiS and β-NiS phases was therefore formed (Fig. 1b). The decomposition temperature was further raised to 350 °C, to gain more insight into the effect of temperature on the phase transformation process. At this temperature, the intensity of the β-NiS peaks was enhanced while that of α-NiS peaks decreased (Fig. 1c). Heating the complex at 400 °C produced the dominant β-NiS phase. However, a meticulous examination of the diffraction pattern reveals the presence of a few extremely low intensity peaks for α-NiS phase. Nonetheless, the ratio between two phases indicates that, 97% of the mixture is composed of the β-NiS phase, and α-NiS occupies only 3%. In an attempt to eliminate the α-NiS phase, complex (**1**) was further heated at 450 and 500 °C, in which a mixture of the two phases was still formed (Figure S2, ESI). The percent of the α-NiS and β-NiS phases at all pyrolysis temperatures are shown in Table S1. It can be observed that, the α-NiS-β-NiS mixture comprising the smallest amount of the α-NiS phase is obtained at 400 °C. The average crystallite sizes were calculated for the particles synthesized at 200 and 400 °C using the Scherrer equation, and were found to be 18.2 and 60.5 nm respectively. These results demonstrate reversibility in the temperature dependent phase behavior normally observed for the two polymorphs, as the α-NiS phase has been obtained at low temperature while the β-NiS phase has been obtained at high temperature^22^. The reversibility might be influenced by the content of Ni and S present in the system at a specified temperature. It has been reported that, the β-NiS phase is formed when the ratio of Ni:S is in the 64:36–67:33 wt% range, and the α-NiS phase is formed when the ratio of Ni:S is in the 63:37–64:34 wt% range^52,53^. The intricate stoichiometric difference between the two phases makes it very challenging to obtain pure phases in the absence of any surface stabilizing reagents. Being sulfur-rich precursors, xanthates are therefore expected to favour the formation of the α-NiS phase even at low temperatures. However, with further increase in temperature, some of the sulfur may be lost owing to its high partial pressure, favoring the formation of the β-NiS phase. Generally, in this case, α-NiS is the kinetically favored phase, while β-NiS is the thermodynamically favored phase^54^. The formation of α-NiS at comparatively low temperature has been observed previously by the thermolysis of a dithiocarbamate complex^54^.

Synthesis of the particles from complex **(2)** produced hexagonal, α-NiS as the only phase at all reaction temperatures (250, 300 and 400 °C) (Figure S3, ESI). The corresponding crystallite sizes were calculated using the Scherer equation and were found to be 30.6, 31.4 and 28.7 nm. No phase transformation was observed in this case. Phase transition in nanomaterials can be influenced by several factors including change in particle size^55,56^, surface properties^57^, as well as temperature^58^. The relatively high thermal stability of the complex and the presence of a long alkyl chain xanthate as a precursor are thought to be the main contributing factors to the observed behavior. For melt synthesis, the ligands left behind after decomposition of the precursor can in turn be used to stabilize/control the growth of the synthesized nanoparticles^45^. Long chain xanthates have been shown to provide better capping than short chain xanthates^42^. Particles synthesized from complex **(2)** are thus expected to be more stable than those synthesized from complex **(1)**. This can restrict the growth of new crystal phases preventing structural phase transitions. The same theory also suggests that the observed phase transition for particles synthesized from complex **(1)** might also be a result of the low stability of the particles.

Henceforth, the NiS samples prepared from complex (**1**) at 200, 300, and 400 °C will be referred to as NISE-1, NISE-2 and NISE-3 respectively, while the sample synthesized from complex (**2**) at 250 °C will be referred to as NISO.

**Figure 2:**
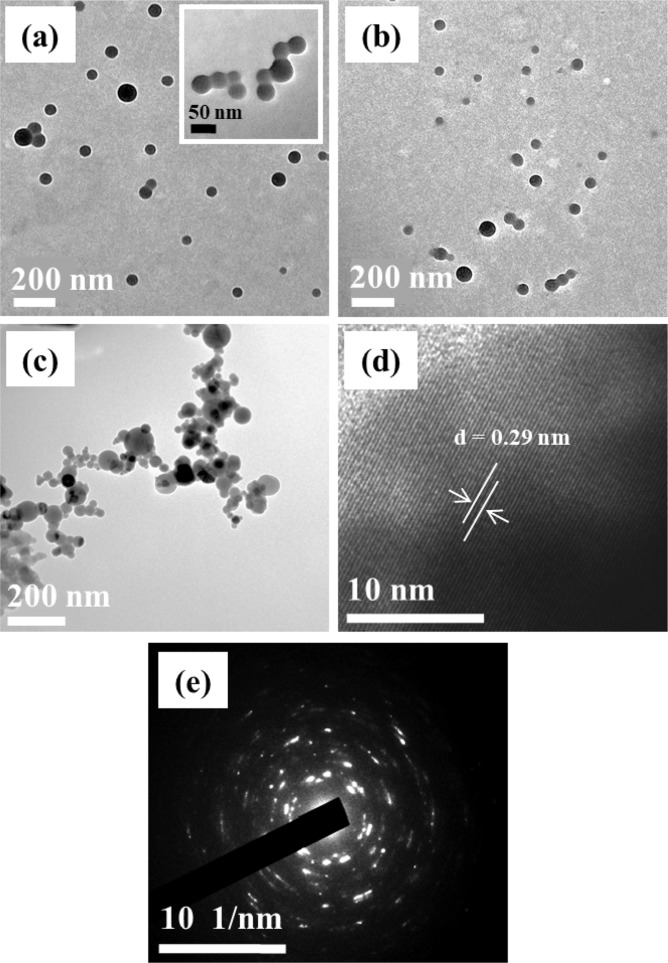
TEM images of NiS nanoparticles synthesized from complex **(1)** at (**a**) 200 °C, (**b**) 300 °C and (**c**) 400 °C. HRTEM (**d**) and SAED (**e**) images of NiS nanoparticles synthesized from complex **(1)** at 200 °C.

TEM analysis of NISE-1, NISE-2 and NISE-3 shows the synthesis of spherically/quasi-spherically shaped particles (Fig. 2). Particle-particle interaction (coalescence) between several neighboring nanospheres can be observed in all three cases (See inset in Fig. 2a). Coalescence in nanoparticles is associated with surface energy reduction^59^. Because of their small dimensions, nanoparticles are normally unstable, highly reactive possessing high surface energy. Coordinating ligands, which are commonly used to stabilize/control the growth of nanoparticles act as a barrier to coalescence^60,61^. In this study, particles synthesized from the short chain xanthate are expected to be either poorly or unpassivated. The observed interaction can thus be explained as an attempt to reduce the high surface energy. Generally, dipole-dipole^61^, Van der Waals, or Columbic^62^ interactions have been identified as the possible forces behind coalescence in nanoparticles. The degree of particle-particle interaction is more prominent for the particles synthesized at 400 °C. The sizes of the particles were found to vary from 32–111 nm for NISE-1, 34–81 nm for NISE-2, and 16–112 nm for NISE-3. The plots showing size distribution of the particles at 200 °C, 300 °C and 400 °C are presented in Figure S4, ESI. It can be seen that, the particles show high polydispersity in terms of size, which can also be explained by the absence of a capping agent and the use of a short chain xanthate.

The representative HRTEM image of particles synthesized at 200 °C shows lattice fringes with a 0.29 nm *d-* spacing which corresponds to the [100] plane of hexagonal NiS (Fig. 2d). The selected area electron diffraction (SAED) image of the particles shows bright spots which substantiate the crystallinity of the material (Fig. 2e). On the contrary, the particles synthesized from complex **(2)** are mainly disc-shaped (Figure S5, ESI). This means that the long alkyl chain does not only contribute to the high thermal stability of the complex but also significantly changes the shape of the synthesized nanoparticles. Unlike the spherical particles prepared from complex **(1)**, no merging or fusion of particles was observed in this case.

**Figure 3:**
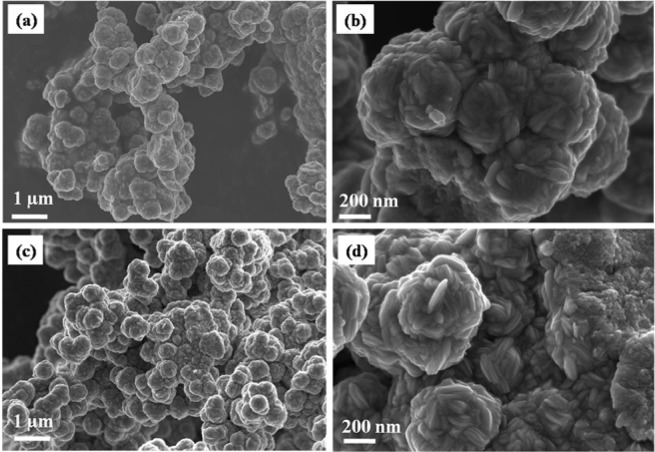
SEM images of NiS nanoparticles synthesized from complex **(1)** at (**a**,**b**) 200 °C, and (**c**,**d**) 400 °C.

SEM analysis was carried out for the nanoparticles synthesized from complex (**1**) at 200 °C (α-NiS) and 400 °C (β-NiS). SEM images of the particles prepared at both temperatures show the synthesis of coalesced spherical nanoparticles, corroborating the TEM observations (Fig. 3). The elemental composition of the two samples was examined by EDX analysis. The corresponding EDX spectra indicate the presence of nickel and sulfur as the only constituents (Figure S6, ESI). The atomic percentage compositions along with the calculated weight percent compositions are displayed in Table S2, ESI. The Ni:S molar ratio in α-NiS is determined to be 0.95:1.05, while that of β-NiS is 0.99:1.01. These values match well with the expected 1:1 nickel to sulfur molar ratio. The calculated weight percentages also agree with the reported values determining the formation of α- or β-NiS phase^52,53^. Elemental mapping of the samples indicate a homogeneous distribution of the two elements in the samples (Figure S7, ESI).

**Figure 4:**
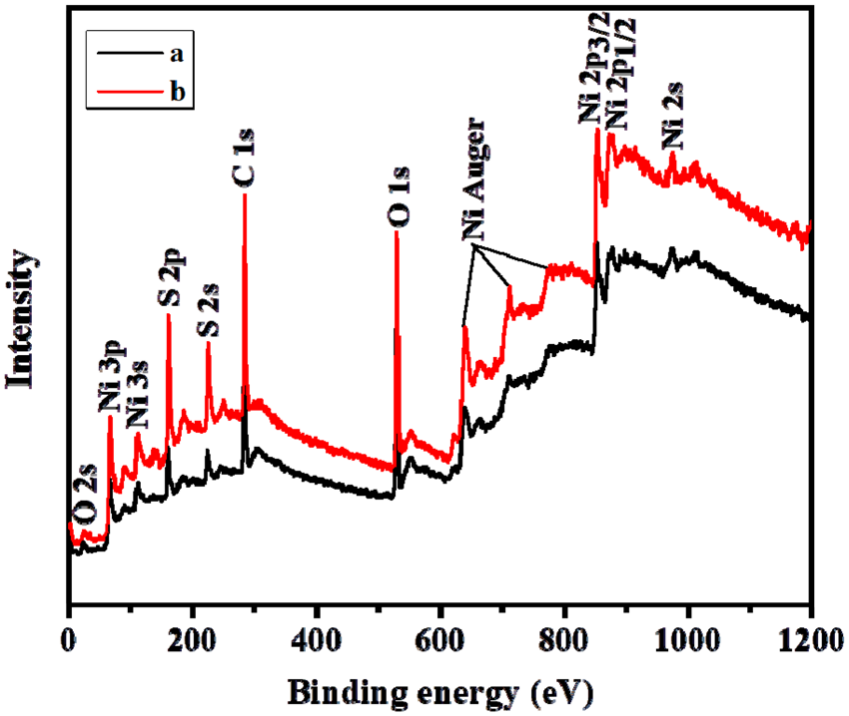
XPS survey spectra of NISE-1 (**a**) and NISO (**b**).

**Figure 5:**
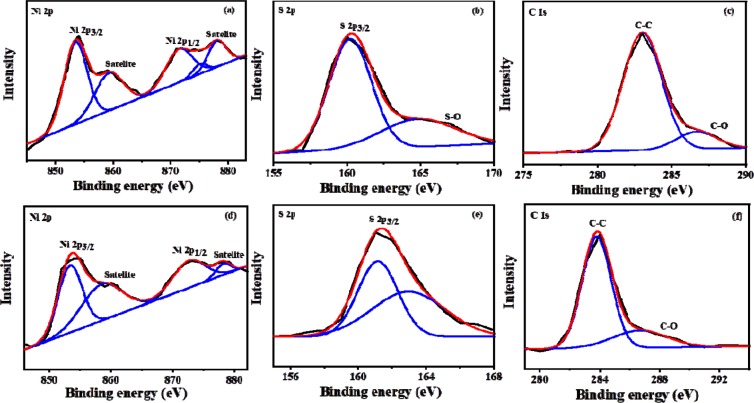
XPS core level spectra of NISE-1 (**a**–**c**) and NISO (**d**–**f**) samples for Ni 2p, S 2p and C1s.

The surface composition and chemical states of NiS were examined by XPS. The analysis was carried out for the NiS sample synthesized from complex **(2)** at 250 °C (NISO), and one representative sample from the short chain xanthate (NISE-1). The survey spectra of both NISE-1 and NISO depict the presence of Ni, S, O and C (Fig. 4). The observed oxygen signal can be ascribed to partial oxidation resulting from NiS exposure to air. The presence of carbon is most likely attributed to the surface ligands generated during decomposition of the precursors^45,63^. The Ni 2p, S 2p, and C 1 s spectral regions of the two NiS samples were fitted by the Gaussian method, and are shown in Fig. 5. The Ni 2p region of NISE-1 displays four peaks. The peaks located at 853.4 and 871.4 eV can be assigned to the Ni 2p_3/2_ and Ni 2p_1/2_ states of Ni^2+^ respectively, whereas those observed at 859.2 and 878 eV are the corresponding satellite peaks (Fig. 5a)^64,65^. For the S 2p spectrum, the peak at 160.8 eV corresponds to S 2p_3/2_, and suggests that the sulfur species in NISE-1 exist as divalent ions (S^2−^). The broad peak at 165.9 eV is associated with the oxidized species of sulfur (SO_x_^2−^) (Fig. 5b)^65–67^. This observation lines up with the oxygen signal displayed at 529.9 eV. The two peaks displayed in the C 1 s spectrum can be attributed to the C-C (283.1 eV) and C-O (286.7 eV) bonds originating from the precursor decomposition products (Fig. 5c). Similar features were observed for NISO, with the S-O bond peak of S 2p being less prominent in this case (Fig. 5d–f). This analysis therefore confirms the synthesis of NiS nanoparticles containing some carbonaceous materials. The higher intensity of the C 1 s peak of NISO suggests that the NiS nanoparticles synthesized from the long chain xanthate contain a high amount of carbonaceous materials as compared to the particles synthesized from the short chain xanthate.

### Electrochemical properties

**Figure 6:**
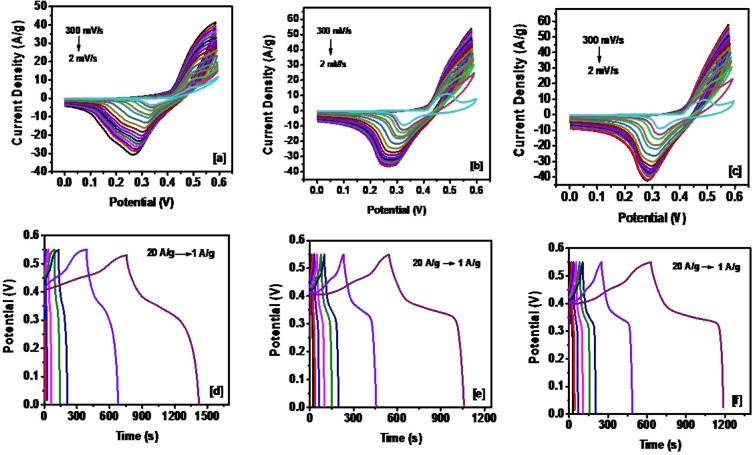
CV curves at various scan rates for NiS synthesized from the complex **(1)** at (**a**) 200 °C, (**b**) 300 °C and (**c**) 400 °C. Galvanostatic charge and discharge curves of NiS synthesized from the complex **(1)** at (**d**) 200 °C, (**e**) 300 °C and (**f**) 400 °C.

**Figure 7:**
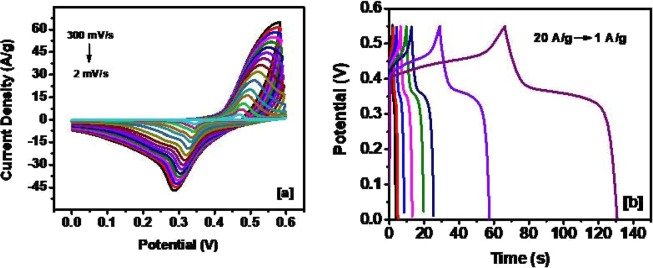
(**a**) CV curves at various scan rates (**b**) Galvanostatic charge and discharge curves, for NiS sample synthesized from the complex **(2)** at 250 °C.

The electrochemical properties of NiS were studied using cyclic voltammetry (CV) and galvanostatic charge-discharge (CD) measurements. The cyclic voltammograms of NiS synthesized from complex **(1)** at various temperatures (200, 300 and 400 °C) are shown in Fig. 6a–c. The area under the CV curves is observed to increase with increase in temperature, indicating improvement in the charge storage capacity. The shape of the CV curves is also shown to be identical even at higher scan rates, signifying high stability in charge transfer. The charge-discharge curves obtained at various current densities are shown in Fig. 6d–f. It was shown that a longer discharge time was observed for α-NiS, which also suggests improvement in charge storage capacity, followed by β-NiS, and the composite showed lower discharge time as compare to pure α-NiS or β-NiS. Figure 7a,b shows the CV and CD curves of the NiS synthesized from complex **(2)**. The identical CV curves may represent a high stability during the process, and the increase in the discharge time with a decrease in the current density may imply an improvement in the storage capacity at lower applied current. It was interesting to note that, despite having a similar phase, the discharge time for α-NiS (NISO) obtained from the decomposition of the octyl xanthate precursor was much shorter (almost 10 times) as compared to the α-NiS (NISE-1) obtained from the ethyl xanthate precursor.

**Figure 8:**
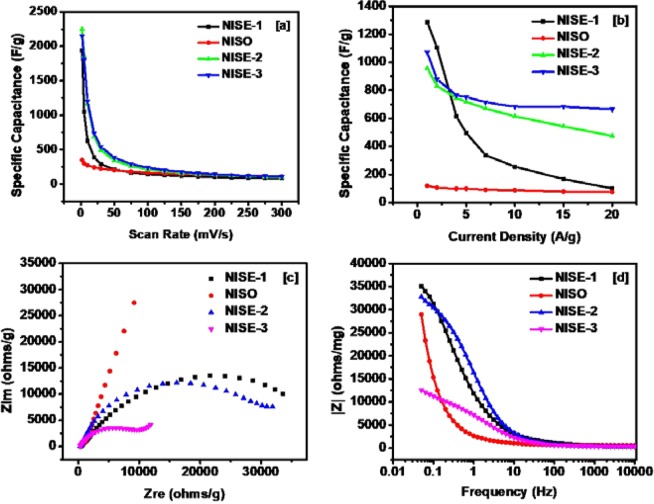
(**a**) Variation of specific capacitance versus scan rate for all synthesized samples, (**b**) Variation of specific capacitance versus applied current density for all synthesized samples, (**c**) Zre vs Zim plots of the devices, (**d**) Variation of impedance as a function of frequency for the devices.

The variation of specific capacitance as a function of scan rate for all synthesized samples is shown in Fig. 8a and summarized in Table 1. The highest specific capacitance of 2,250 F/g was observed for NISE-2 at the lower scan rate (2 mV/s). This value is much superior, for example, to the high capacitance obtained for Ni_3_S_2_ synthesized by Chen *et al*., which is 1,325 F/g at a scan rate of 2 mV/s^68^. This superior performance of NISE-2 can be attributed to the existence of both the α-NiS, and β-NiS phases in a single system. It is known that, for a mixed phase system, the crystal lattice mismatch, resulting from the two different crystal structures, prevents the growth of a defect-free material^27,69,70^. In some cases, this has been demonstrated to have an impact on the electrochemical properties of the material. For instance, Wang *et al*. synthesized a dual-phase system of NiS-Ni_7_S_6_ which showed high capacity as an electrode material for rechargeable lithium batteries^71^. Similarly, Idris *et al*. synthesized a binary α-NiS-β-NiS system which also showed improved lithium storage performance^27^. In the α-NiS phase, nickel atoms exist in a six coordination system with the sulfur atoms, while in the β-NiS phase each nickel atom is coordinated to five sulfur atoms^72^. Their binding energies are however comparable^72^. Formation of a composite of the two phases will therefore result in a mixed coordination system which can in turn affect the electrochemical properties of the material.

**Table 1:** The specific capacitance of synthesized samples at various scan rates.

Samples	Specific capacitance (F/g) at 2 mV/s	Specific capacitance (F/g) at 50 mV/s	Specific capacitance (F/g) at 100 mV/s	Specific capacitance (F/g) at 300 mV/s
NISE-1	1,940	210	144	82
NISO	349	199	165	111
NISE-2	2,250	341	213	99
NISE-3	2,150	382	238	107

Variation of the specific capacitance versus current density for all samples is shown in Fig. 8b. As observed, a high specific capacitance of 1,287 F/g was achieved for NISE-1 at a current density of 1 A/g. This value can be compared to the highest specific capacitance of 1,077 F/g at 5 A/g, achieved for Ni_3_S_2_-NiS nanowires made by Zang *et al*.^73^. The value is also higher or comparable to NiS and other commonly used storage materials synthesized by other methods (Table S3, ESI). The decrease in specific capacitance with increasing current density could be due to insufficient time for the electrolyte ions to diffuse into the inner pores. In contrast to NISE-1, the capacitive behavior of NISO was observed to be much lower regardless of the fact that both samples have similar crystal phases. Among other factors, the charge storage behavior can also be affected by the nature and concentration of the surfactants^74–76^. An optimum amount of surfactant results in enhanced supercapacitance, whereas a higher concentration or densely populated surface may have a detrimental effect on the charge storage. It is a well-known observation that the decomposition of the xanthate complexes with long alkyl chain results in the formation of size controlled nanomaterials via a self-capping phenomenon. The solventless thermolysis of the long chain xanthate (complex (**2**)) is therefore expected to produce self-capped NiS nanoparticles. To confirm this presumption, IR analysis of both NISE-1 and NISO was carried out and the results are shown in Figures S8, ESI. Unlike NISE-1, the IR spectrum of NISO shows the presence of alkane C-H stretch around 2900 cm^−1^, and generally resembles that of the complex. This implies that the surface of NISO is indeed passivated by the ligand produced during decomposition of the precursor. The presence of residual carbon on the surface of NISO may therefore act as an insulating layer, diminishing its performance. Unlike NISO, the surface of NISE-1 has much lower carbon content, and hence charge accumulation is substantially higher.

**Figure 9:**
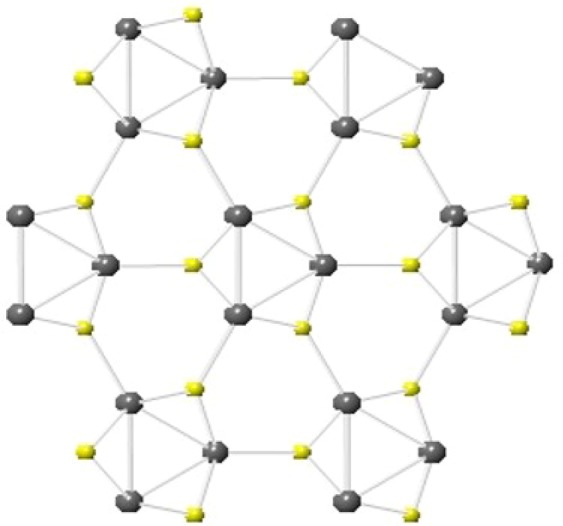
Crystal structure of NiS millerite phase viewed along [001] plane. The image was drawn by using the licensed CrystalMaker software (version 9.2.9f1 (665)) (http://crystalmaker.com/).

**Figure 10:**
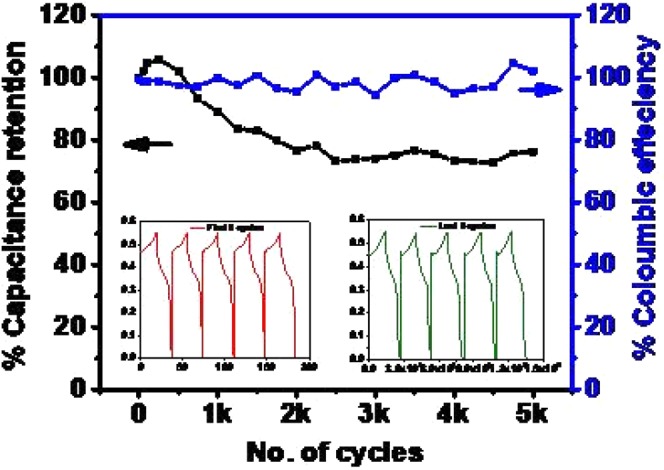
Capacitance retention and Coulombic efficiency as a function of a number of cycles for NISE-1.

The effect of temperature on the electrochemical behavior of the supercapacitor was investigated by electrochemical impedance spectroscopy (EIS). The variation of real and imaginary impedance of all the samples is shown in Fig. 8c, and the variation of impedance as a function of frequency for all the samples is illustrated in Fig. 8d. Excellent electrical conductivity of the device was observed for NISE-3, with a very small cell resistance. The high electrical conductivity is attributed to the metallic nature associated with the β-NiS phase^28,77^. The metal-like behavior of millerite can be explained by its crystal structure as shown in Fig. 9. The structure indicates an arrangement of Ni atoms in a three-member cyclic ring and comprises of Ni_3_S_9_ clusters. There is no Ni-Ni bonding between adjacent cyclic Ni_3_ rings, and are linked by S atoms. The coordinating sulfur atoms further interconnects the Ni_3_S_9_ clusters and develop a framework of Ni-S. The high conductivity and electron transport can be attributed to Ni(d)-S(p) charge transfer interactions. The electron transfer between the adjacent metallic three membered Ni rings can take place by the hopping of electrons from d-orbitals of one Ni_3_ ring to other via the p-type orbitals of the coordinating S atoms. This electron transport results in high conductivity and explains the increased charge mobility and hence the lowest resistance. The highest resistance was observed for NISO, for which almost a straight line was observed, which further confirms the insulating nature, despite existing in the hexagonal α-NiS phase. Further, the stability of NISE-1 was investigated for applications as a high-performance supercapacitor device. In Fig. 10, the performance of the sample for 6,000 cycles is shown. An excellent result, reaching 100% of Coulombic efficiency and 79% of capacitance retention was observed, which is higher as compared to 76.3% for Ni_3_S_2_-NiS nanowires from Zang’s synthesis^73^. This suggests that the sample provides high performance for energy storage devices.

**Figure 11:**
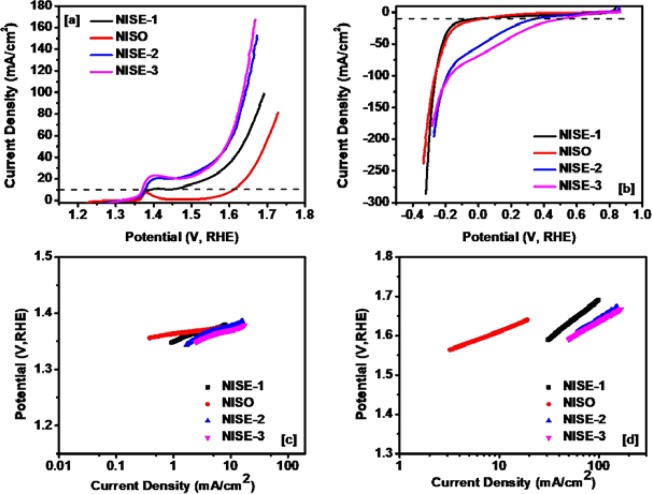
Polarization curves for (**a**) OER, (**b**) HER, and Tafel slopes for (**c**) OER, (**d**) HER.

The electrocatalytic performance of NiS samples synthesized from complexes **(1)** and **(2)** as OER and HER electrocatalysts was investigated using linear sweep voltammetry (LSV). The polarization curves in OER for all samples are shown in Fig. 11a. The overpotentials for the samples were found to be 139 mV for NISE-3, 147 mV for NISE-2, 161 mV for NISE-1, and 380 mV for NISO at a current density of 10 mA/cm^2^. Xiao *et al*. have synthesized NiS/Ni_2_P in carbon cloth, which provides an overpotential of 265 mV for the OER to reach a current density of 20 mA/cm^2^ ^30^. According to Chen *et al*., NiS grown on stainless steel exhibits an overpotential of 297 mV at 11 mA/cm^2^, with a Tafel slope of 47 mV/dec^78^. As shown in our work, NISE-3 displayed the lowest overpotential (139 mV) compared to the other studied samples and other reports in the literature, ensuring best energy generation applications. The catalytic performance was also much higher as compared to the other commonly used binary/ternary oxide-based and/or Ni-based state of the art OER catalysts. A detailed comparison of the best OER activity achieved in this work with some previously reported Ni-based electrocatalysts is given in Table S4, ESI. The OER activity of the NiS samples was also compared with that of RuO_2_ and Pt electrocatalysts, in which NISE-3 demonstrated superior performance (NISE-3 > NISE-2 > NISE−1 > RuO_2_ > NISO > Pt (Figure S9, ESI).

The Tafel slope indicates the reaction kinetics for the different catalysts. For NISE-3, which presents the lowest overpotential, the Tafel slope is only 32 mV/dec, showing a fast and efficient process (Fig. 11c). The polarization curves for HER are provided in Fig. 11b, indicating best result for NISE-3. For NiS/Ni_3_S_4_ synthesized by Qin *et al*., the HER activity reached a low overpotential of 221 mV at 10 mA/cm^2^, which is comparable with our results^79^, whereas, it is much superior as compared to NiS (474 mV at 10 mA/cm^2^), NiS_2_ (454 mV at 10 mA/cm^2^), Ni_3_S_2_ phase (335 mV at 10 mA/cm^2^) and Ni_3_S_2_-CNTs composites (480 mV at 10 mA/cm^2^)^25,80^. A detailed comparison is given in Table S5, ESI. The reaction kinetics for all samples in HER is shown in Fig. 11d. Generally, the millerite, β-NiS phase has shown comparatively, much higher catalytic efficiency as compared to the hexagonal α-NiS phase. It can be seen that, by the introduction of the β-phase into an α-phase, the catalytic efficiency can be drastically enhanced.

The series resistance of the synthesized samples was analyzed using EIS. The frequency versus impedance of the NiS synthesized from complex **(1)** at various temperatures (200, 300 and 400 °C) is shown in Figure S10a–c, ESI. The lowest charge transfer resistance was achieved, as expected, for the sample synthesized at 400 °C, which means more rapid electrochemical reaction kinetics for metal-like β-NiS. The Nyquist plot showed in Figure S10d–f, ESI appear as small semicircles, indicating high mobility of ions that increase the conductivity. For the NiS sample synthesized from complex **(2)**, the frequency versus impedance is shown in Figure S11. The lowest resistance achieved was about 7 Ω, which is higher than the resistances achieved for all the samples obtained from complex **(1)**. Similarly, the semicircle generated in the sample from complex **(2)** is bigger than all semicircles in samples from complex **(1)** (Figure S11b, ESI). Hence, it can be inferred that the samples synthesized from the xanthate complexes with short alkyl chain have higher conductivity than the sample from the complex containing the longer alkyl chain.

**Figure 12:**
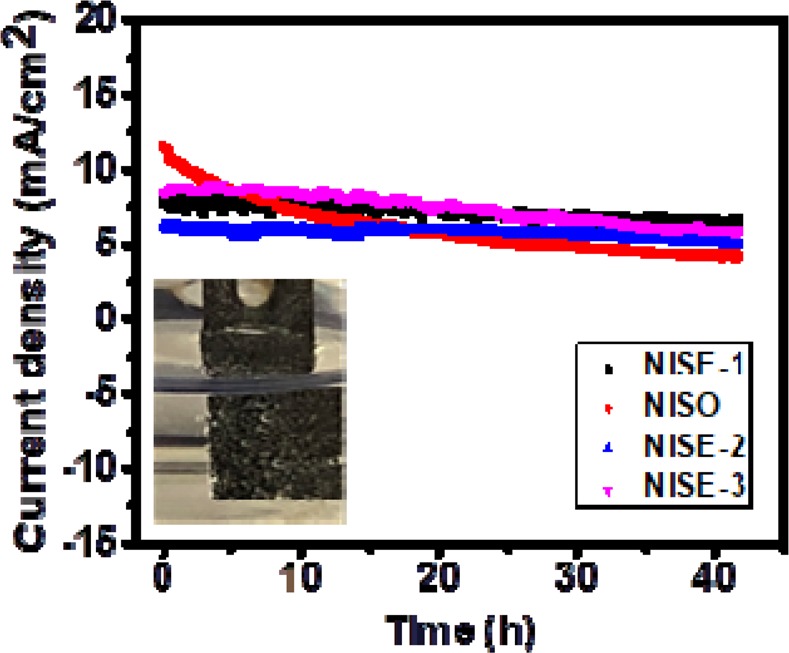
Stability performance of the electrodes using chronoamperometry (inset figure shows photographic image of oxygen evolution during this experiment from the NiS electrode).

The catalytic performance was also tested using the chronoamperometric test. As observed from Fig. 12, the current density of all samples only decreases slightly after 40 hours of testing. From the overall measurements, it is concluded that samples exhibit a stable and durable catalytic performance and can be used as an efficient OER and HER catalytic electrode.

## Conclusion

In conclusion, a facile and potentially scalable route has been used to prepare highly crystalline and phase pure nickel sulfide. The phase can be selectively tuned to α-NiS, β-NiS or α-NiS-β-NiS composite, by simply varying the temperature. The phase and morphology of the synthesized product is significantly affected by the alkyl chain length of the precursor. The electrochemical investigation of the samples indicates that, millerite NiS is comparatively better for both energy storage and energy generation applications. Similarly, the electrochemical properties of the α-NiS phase can be considerably enhanced by the introduction of the β-NiS phase. The highest specific capacitance at 2 mV/s, was observed for the composite (2,250 F/g), which was slightly higher than that observed for the β-NiS phase (2,150 F/g). Similarly, HER and OER were observed to be much higher for the millerite phase as compared to hexagonal NiS. The metal like nature of the millerite phase offers a very low charge transfer resistance, which may lead to better electrochemical properties of the phase. It was also observed that the presence of ligand/carbonaceous material on the surface of NiS can result in drastic reduction of the electrochemical efficiency. The uncapped samples synthesized using short chain xanthate complex showed great potential for both energy generation and energy storage applications.

## Supplementary information

Supplementary Information.
