# Supplementary material for: Direct solvent free synthesis of bare α-NiS, β-NiS and α-β-NiS composite as excellent electrocatalysts: Effect of self-capping on supercapacitance and overall water splitting activity
Source: Sci Rep. 2020 Feb 24;10:3260. doi: 10.1038/s41598-020-59714-9 (PMC7039904; doi:10.1038/s41598-020-59714-9)
Supplement: Supplementary file 1 — Supplementary Information. [file 41598_2020_59714_MOESM1_ESM.doc]

**Electronic Supplementary Information**

**Direct solvent free synthesis of bare α-NiS, β-NiS and α-β-NiS composite as excellent electrocatalysts: Effect of self-capping on supercapacitance and overall water splitting activity**

Ginena B. Shombe,^a^ Malik D. Khan,^a^* Camila Zequine,^b^ Chen Zhao,^b^ Ram K. Gupta,^b^ Neerish Revaprasadu ^a^*

^a^Department of Chemistry, University of Zululand, Private Bag X1001, KwaDlangezwa 3880, South Africa.

^b^Department of Chemistry, Pittsburg State University, Pittsburg, KS 66762, USA.


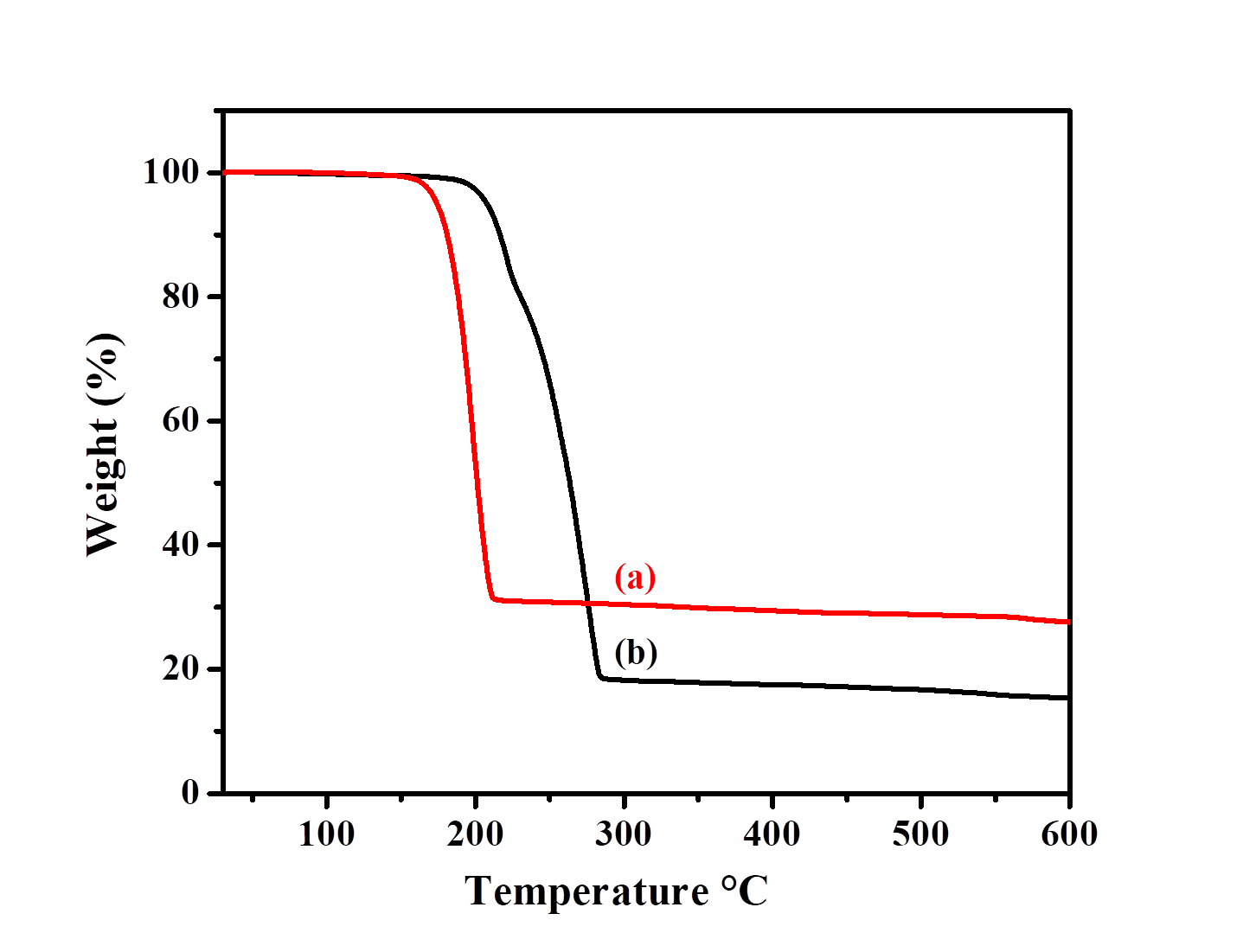


**Figure S1.** TGA plots of complexes **(1)** (a), and **(2)** (b).


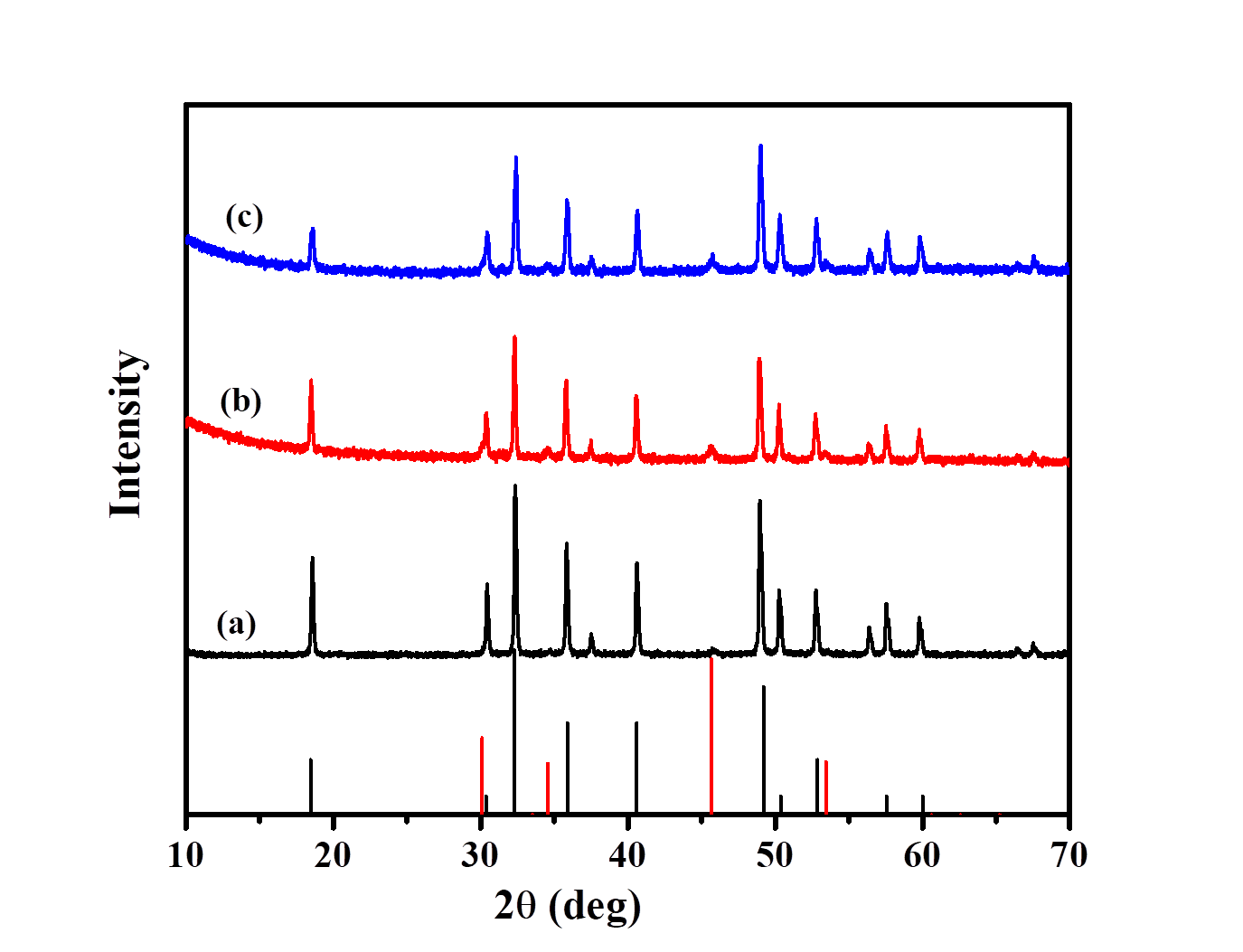


**Figure S2.** p-XRD patterns of NiS nanoparticles synthesized from complex **(1)** at 400 °C (a), 450 °C (b) and 500 °C (c).


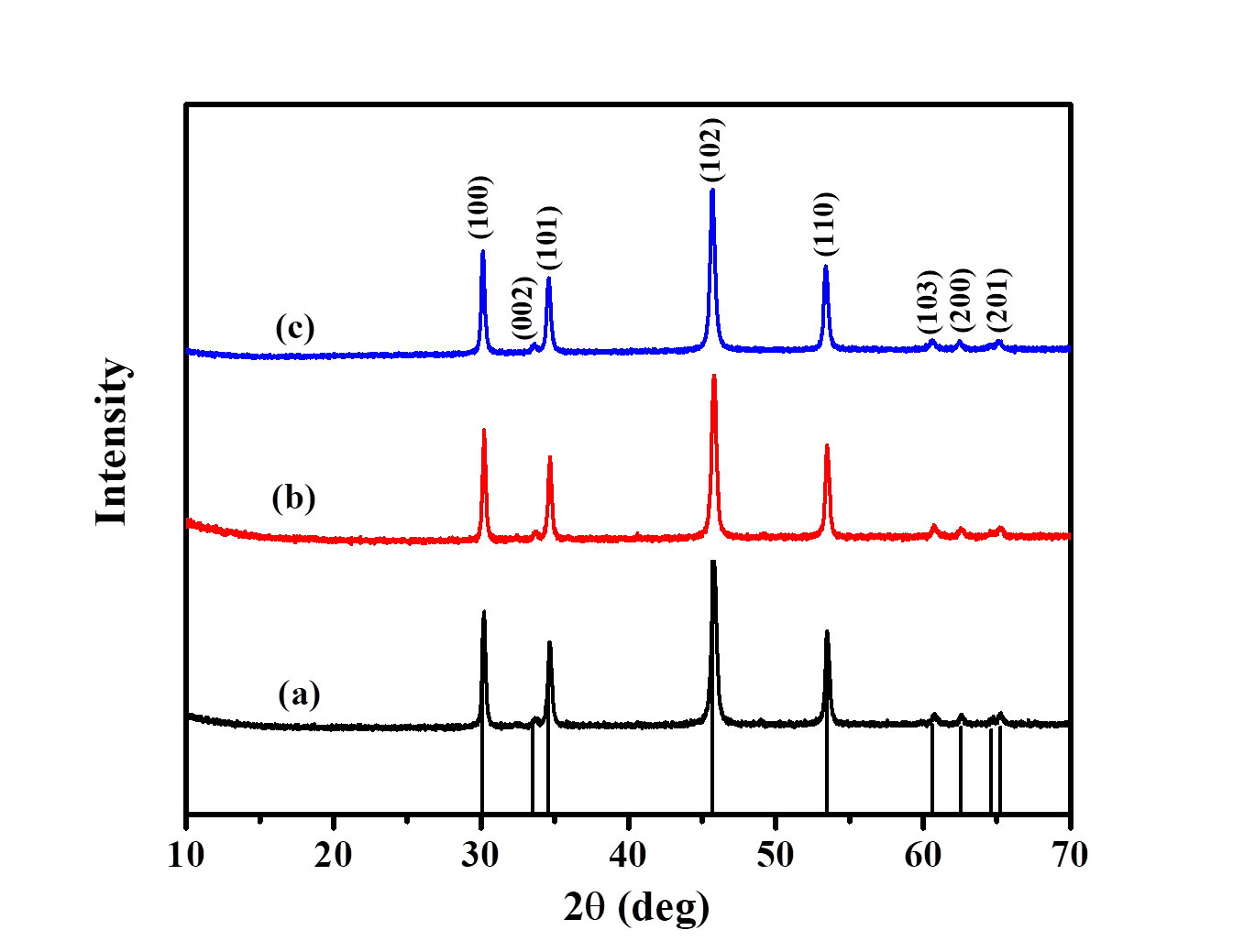


**Figure S3.** p-XRD patterns of NiS nanoparticles synthesized from complex **(2)** at (a) 250 °C, (b) 300 °C and (c) 400 °C.

**
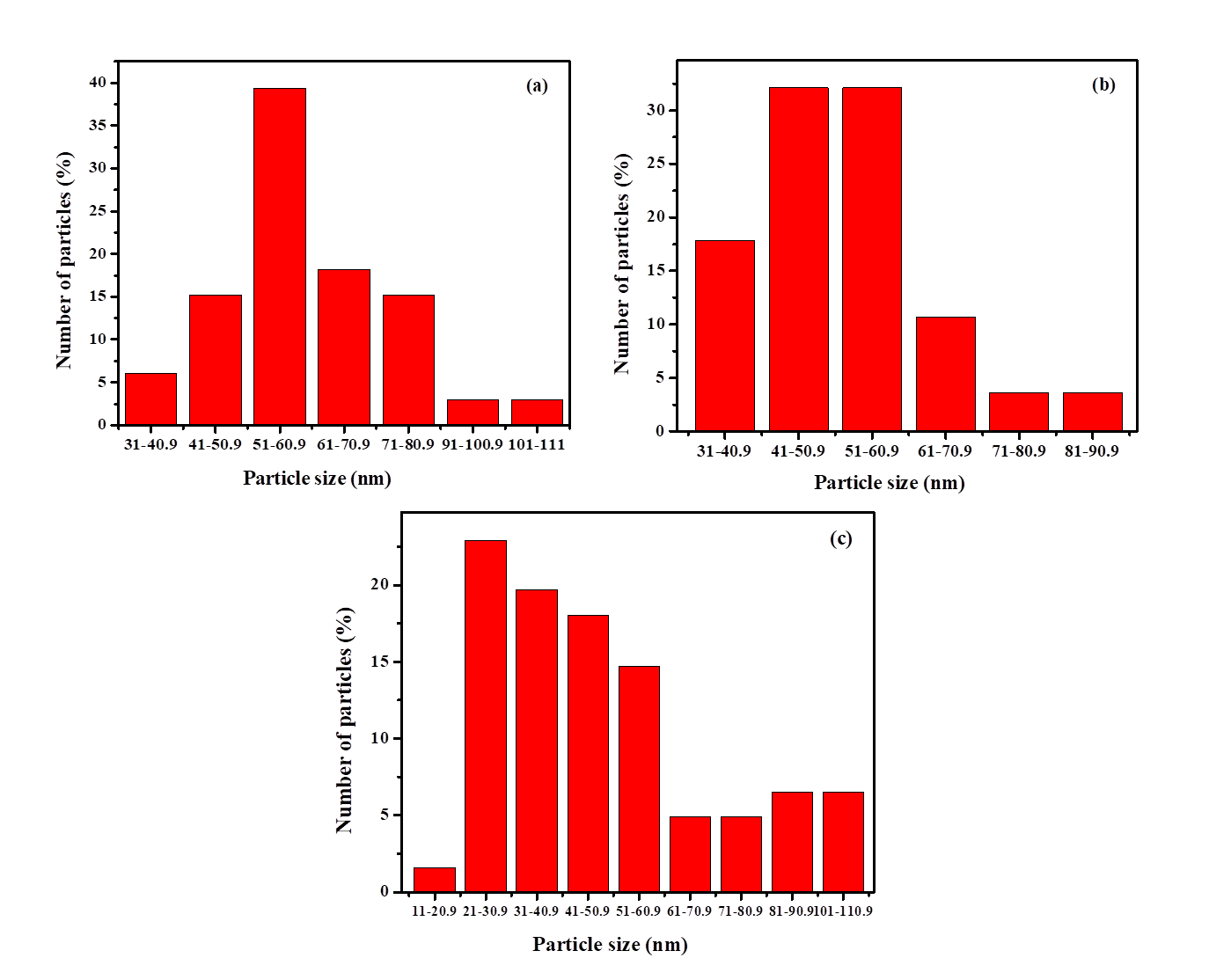
**

**Figure S4.** Size distribution of NiS nanoparticles synthesized from complex **(1)** at (a) 200 °C, (b) 300 °C and (c) 400 °C.


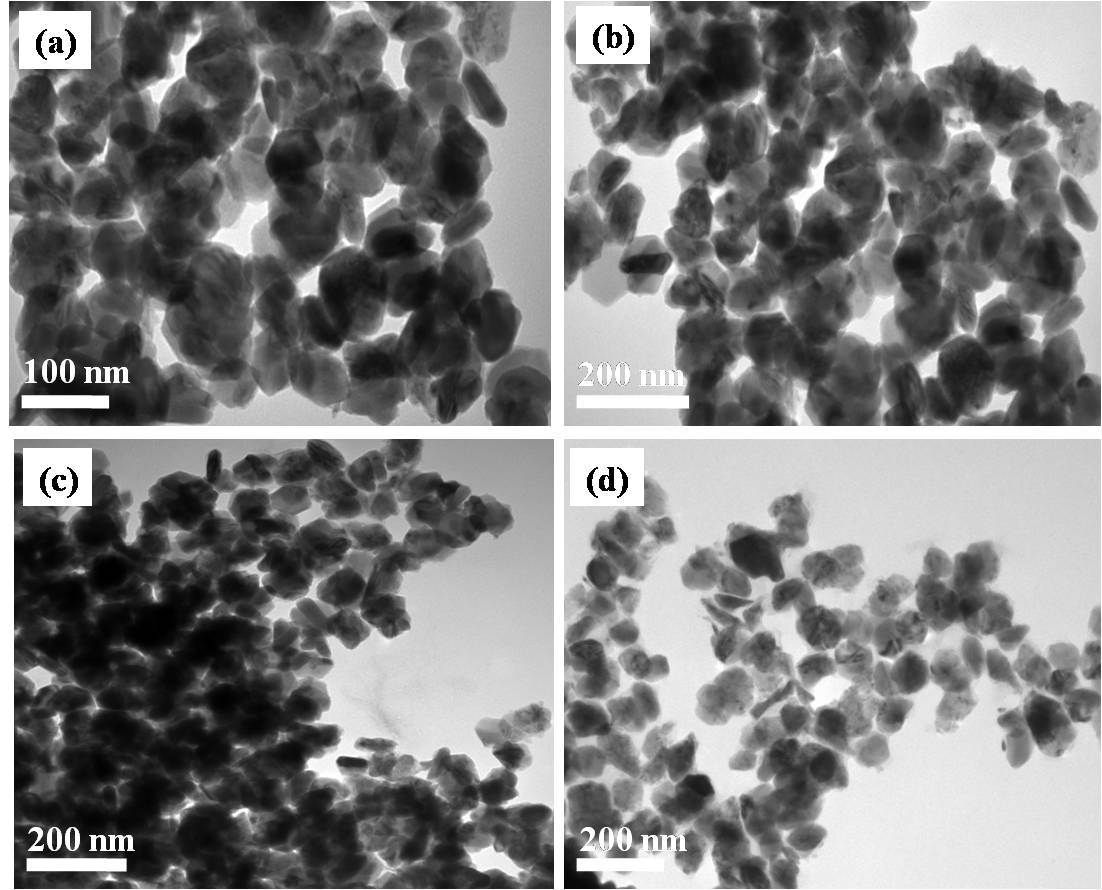


**Figure S5.** TEM images of NiS nanoparticles synthesized from complex **(2)** at (a-b) 250 ºC, (c) 300 ºC and (d) 400 ºC.


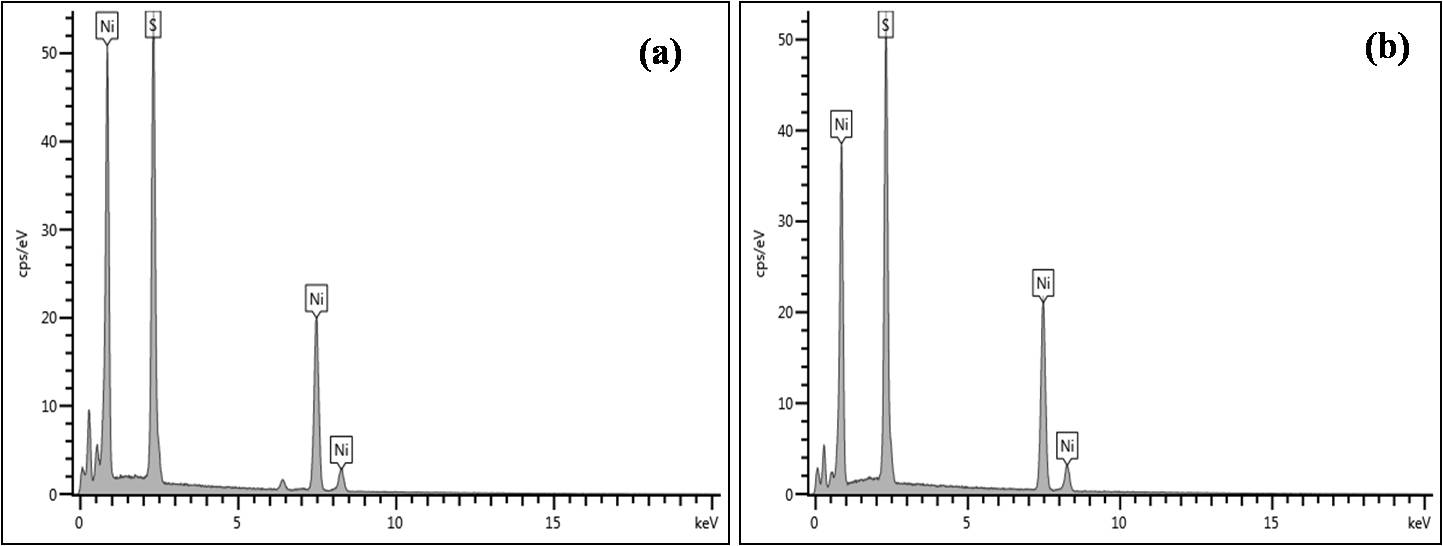


**Figure S6.** EDX spectra of NiS nanoparticles synthesized from complex **(1)** at (a) 200 ºC and (b) 400 ºC.


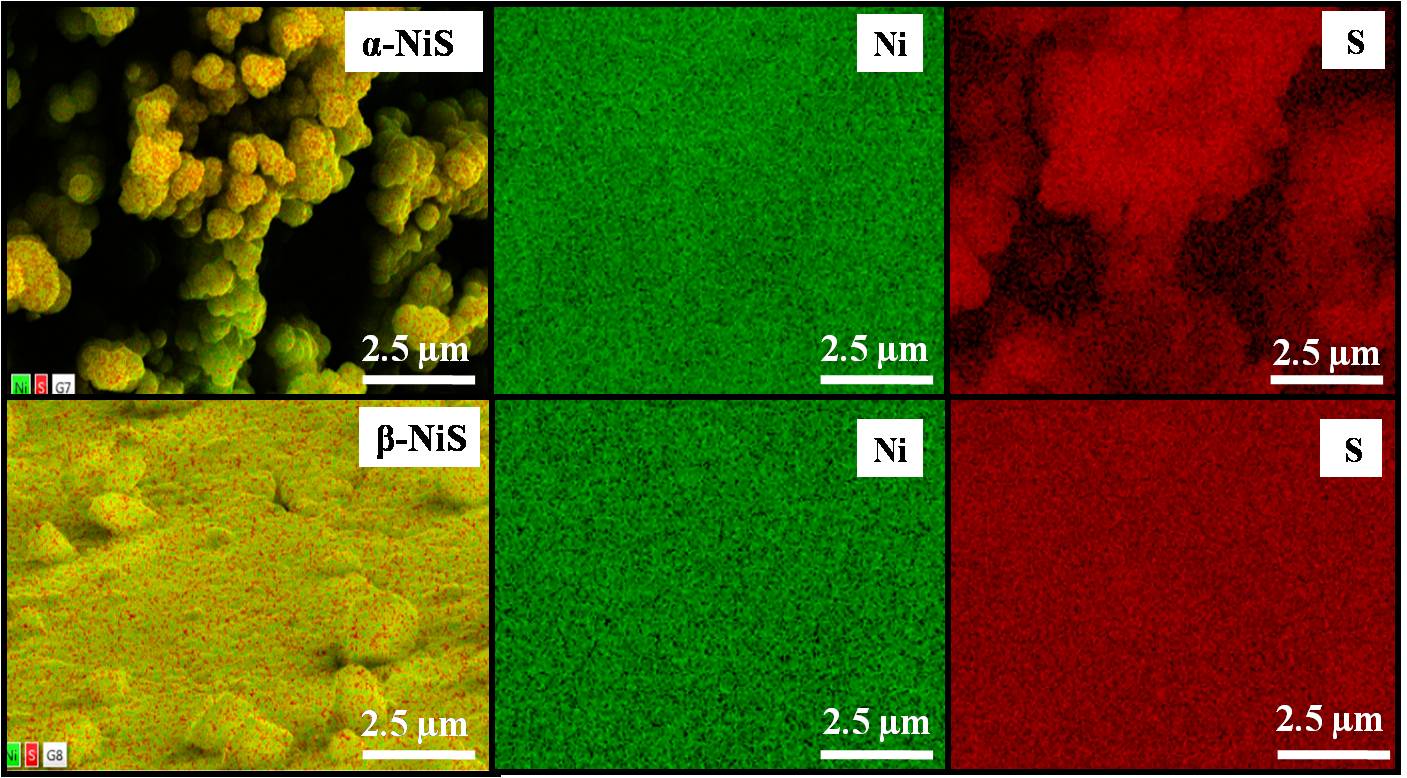


**Figure S7.** Distribution of Ni and S in α- and β-NiS nanoparticles synthesized from complex **(1)**.


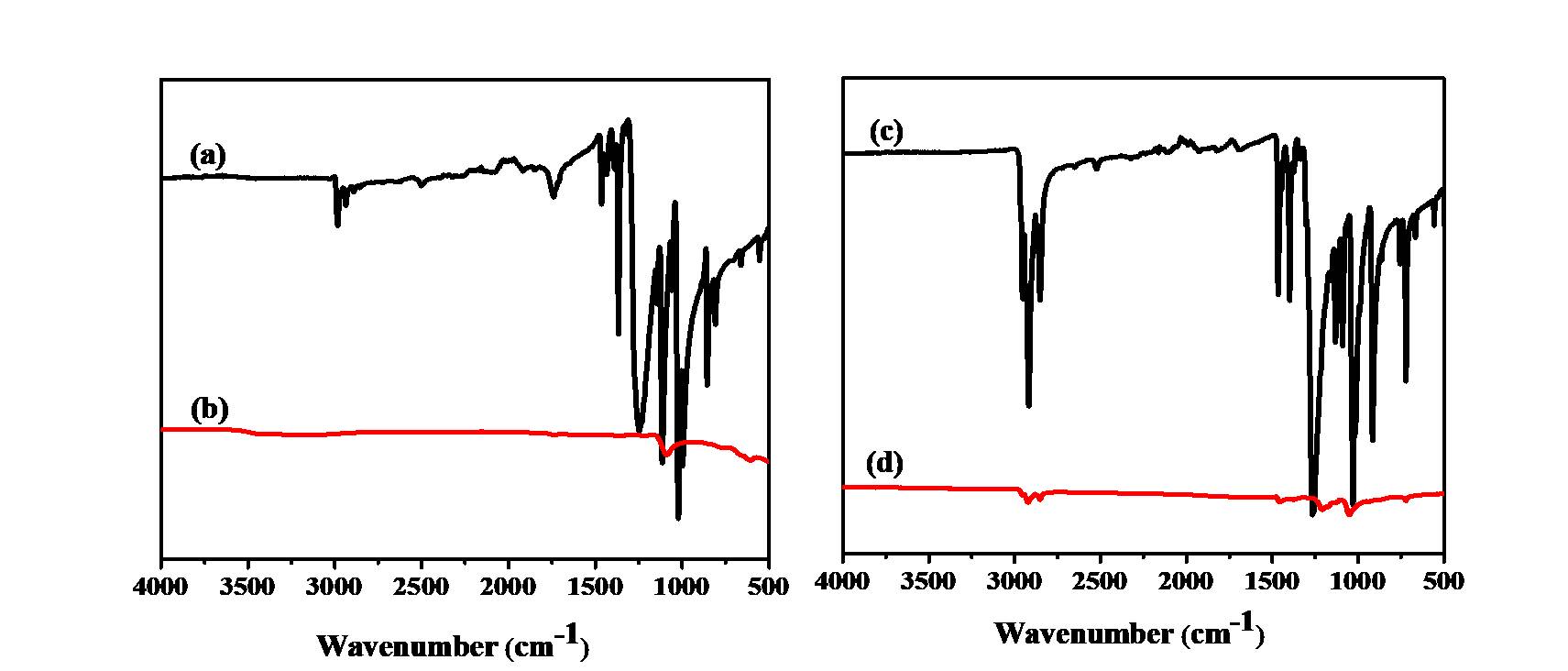


**Figure S8.** IR spectra of (a) complex (**1**), (b) NISE-1, (c) complex (**2**), and (d) NISO.


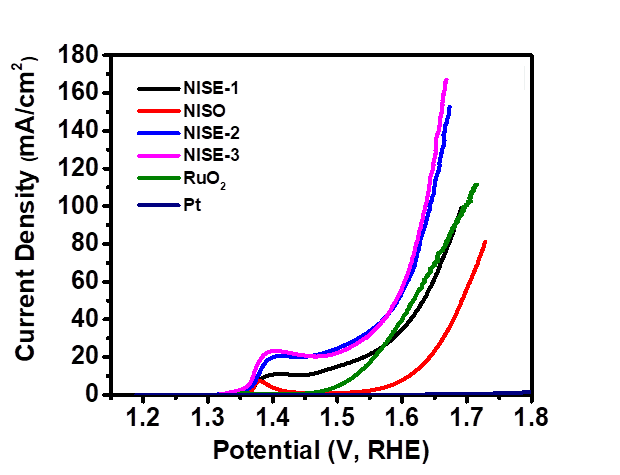


**Figure S9.** A comparison of OER activity of the NiS samples with that of RuO_2_ and Pt.


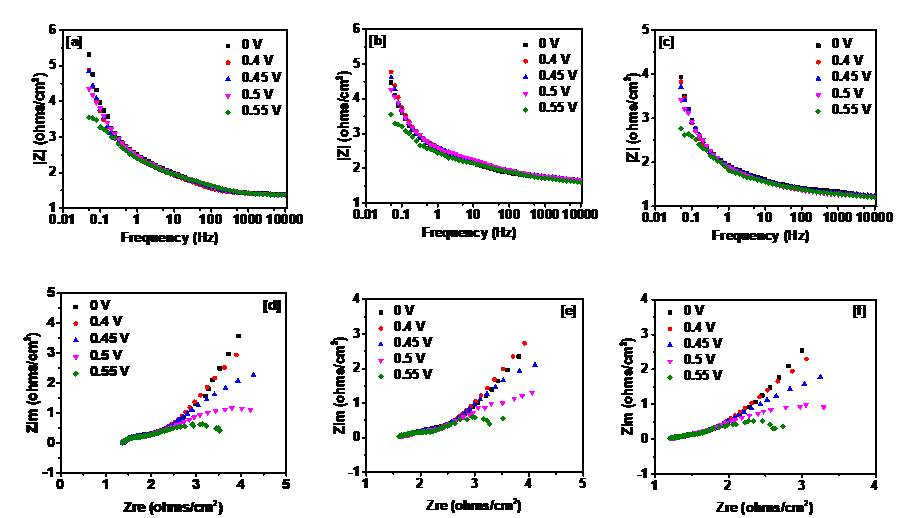


**Figure S10.** (a-c) |Z| vs. frequency plots and (d-f) Zreal vs. Zimg plots at various potentials, for NiS samples synthesized from complex **(1)** at (a, d) 200 °C, (b, e) 300 °C and (c, f) 400 °C.


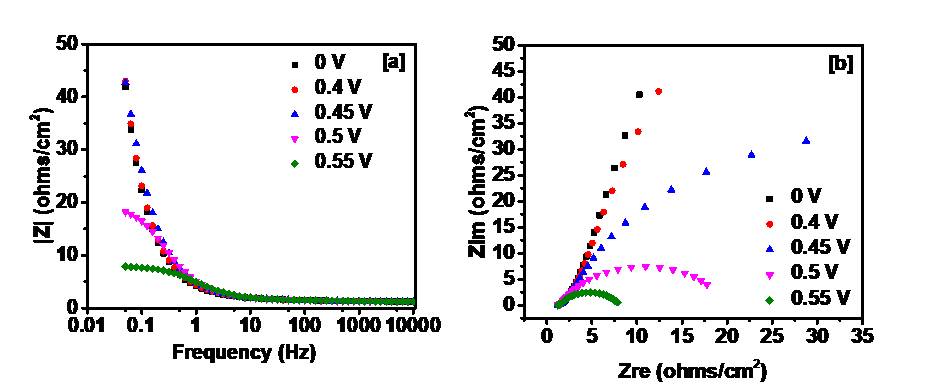


**Figure S11.** (a) |Z| vs. frequency plots and (b) Zreal vs. Zimg plots at various potentials, for NiS sample synthesized from complex **(2)** at 250 °C.

**Table S1.** Percentage of α-NiS and β-NiS at different thermolysis temperatures.

| **Thermolysis temperature (°C)** | **Percentage of α-NiS phase** | **Percentage β-NiS phase** |
| --- | --- | --- |
| 200 | 100 | - |
| 300 | 91 | 9 |
| 350 | 25 | 75 |
| 400 | 3 | 97 |
| 450 | 6 | 94 |
| 500 | 17 | 83 |

**Table S2.** Atomic and weight percentage compositions of α- and β-NiS nanoparticles synthesized from complex **(1)**.

| **Sample** | **At% Composition** | **Wt% Composition** |
| --- | --- | --- |
| α-NiS | Ni: 47.43, S: 52.57 | Ni: 62.2, S: 37.8 |
| β-NiS | Ni: 49.99, S: 50.01 | Ni: 64.1, S: 35.9 |

**Table S3.** Comparison of electrochemical supercapacitor performance of synthesized NiS with other reports.

| **Material** | **Method** | **Specific capacitance (F/g)** | **Current density** | **Reference** |
| --- | --- | --- | --- | --- |
| β-NiS | Template assisted hydrothermal | 668 | 1 A/g | [^1^](#_ENREF_1) |
| β-NiS/GO | Hydrothermal | 109 | 2.5 A/g | [^2^](#_ENREF_2) |
| β-NiS | Chemical bath deposition | 524 | 1 mA/cm^2^ | [^3^](#_ENREF_3) |
| β-NiS/rGO | Hydrothermal | 715 | 1 A/g | [^4^](#_ENREF_4) |
| α-NiS | Hydrothermal | 717 | 0.6 A/g | [^5^](#_ENREF_5) |
| α-NiS | Hydrothermal | 562 | 0.6 A/g | [^6^](#_ENREF_6) |
| β-NiS | Hydrothermal | 501 | 0.6 A/g | [^6^](#_ENREF_6) |
| NiO nanowires | Nano-seed catalyzing mechanism | 180 | 0.5 A/g | [^7^](#_ENREF_7) |
| NiO/rGO | Electrophoretic and chemical bath deposition | 400 | 2 A/g | [^8^](#_ENREF_8) |
| NiO |  | 309 | 1 A/g | [^9^](#_ENREF_9) |
| NiO hollow spheres | Chemical bath deposition | 287 | 1 A/g | [^10^](#_ENREF_10) |
| MoS_2_ | Hydrothermal | 168 | 1 A/g | [^11^](#_ENREF_11) |
| β-MnO_2_ | Hydrothermal | 453 | 0.5 A/g | [^12^](#_ENREF_12) |
| **α-NiS** | **Solventless** | **1287** | **1 A/g** | **This work** |
| **β-NiS** | **Solventless** | **1085** | **1 A/g** | **This work** |

**Table S4.** Comparison of OER performance of synthesized NiS with other reports.

| **Catalyst** | **Overpotential (mV) at 10 mA/cm^2^** | **Tafel Slope (mV/decade)** | **Reference** |
| --- | --- | --- | --- |
| **β-NiS** | **139** | **32** | **This work** |
| NiS micro architectures | 320 | 59 | [^13^](#_ENREF_13) |
| NiS@N/S-C nanocomposites | 417 | 48 | [^14^](#_ENREF_14) |
| Ni_3_S_2_ nanostructures | 217 | 163 | [^15^](#_ENREF_15) |
| Ni_3_S_2_ NWs/Ni | 317 | 84.8 | [^16^](#_ENREF_16) |
| Ni_3_S_2_ nanorods/Ni foam | 187 | 159.3 | [^17^](#_ENREF_17) |
| MoS_2_/Ni_3_S_2_ Heterostructures | 218 | 83 | [^18^](#_ENREF_18) |
| NiO_x_ nanoparticles | 330 | 54 | [^19^](#_ENREF_19) |
| Ni-P/CF | 325 | 120 | [^20^](#_ENREF_20) |
| **NiCo_2_O_4_ core-shell nanowires** | 320 | 63.1 | [^21^](#_ENREF_21) |
| Ni_x_Fe_1-x_Se_2_ | 195 | 28 | [^22^](#_ENREF_22) |

**Table S5. Comparison of HER performance of synthesized NiS with other reports.**

| **Catalyst** | **Overpotential (mV) at 10 mA/cm^2^** | **Tafel Slope (mV/decade)** | **Reference** |
| --- | --- | --- | --- |
| β-NiS nanocrystals | 186 at 2.4 × 10^−6^ A cm^−2^ | 51.2 | [^23^](#_ENREF_23) |
| Ni_3_S_2_ | 335 mV at 10 mA cm^−2^ | 97 | [^24^](#_ENREF_24) |
| NiS_2_ | 454 | 128 | [^24^](#_ENREF_24) |
| NiS_2_ Micro-Architecture | 174 at 10 mA cm^−2^ | 63 | [^13^](#_ENREF_13) |
| NiS | 474 mV | 124 | [^24^](#_ENREF_24) |
| NiS_2_ nanosheets array on carbon cloth (NiS_2_ NA/CC) | 193 and 243 mV | 69 | [^25^](#_ENREF_25) |
| NiS_2_ nanowires |  | 83.5 | [^26^](#_ENREF_26) |
| Ni_2.3%-_CoS_2_/CC | 231 at 100 mA cm^−2^ | 106 | [^27^](#_ENREF_27) |
| Ni_3_S_2_/Ni foam | 220 at 10 mA cm^−2^ | 108 | [^28^](#_ENREF_28) |
| NiS_2_/rGO | 200 at 10 mA cm^−2^ | 52 | [^29^](#_ENREF_29) |
| Hierarchically porous Ni_3_S_2_ nanostructures | 200 at 10 mA cm^−2^ | 107 | [^15^](#_ENREF_15) |
| NiS/Ni foam | 158 mV at 20 mA cm^−2^ | 83 | [^30^](#_ENREF_30) |

**References**

1 Yu, X. Y. *et al.* General Formation of MS (M= Ni, Cu, Mn) Box‐in‐Box Hollow Structures with Enhanced Pseudocapacitive Properties. *Advanced Functional Materials* **24**, 7440-7446 (2014).

2 Prusty, B., Adhikary, M. & Das, C. Synthesis of NiS anchored graphenenanocomposites: High performance supercapacitor electrode material. *Int. J. Curr. Res* **6**, 7448-7452 (2014).

3 Patil, A. *et al.* Ultrathin nickel sulfide nano-flames as an electrode for high performance supercapacitor; comparison of symmetric FSS-SCs and electrochemical SCs device. *RSC Advances* **6**, 68388-68401 (2016).

4 Yang, J. *et al.* Electrochemical performances investigation of NiS/rGO composite as electrode material for supercapacitors. *Nano Energy* **5**, 74-81 (2014).

5 Wei, C. *et al.* Comparison of NiS 2 and α-NiS hollow spheres for supercapacitors, non-enzymatic glucose sensors and water treatment. *Dalton Transactions* **44**, 17278-17285 (2015).

6 Wei, C. *et al.* NiS Hollow Spheres for High‐Performance Supercapacitors and Non‐Enzymatic Glucose Sensors. *Chemistry–An Asian Journal* **10**, 679-686 (2015).

7 Pang, H., Lu, Q., Zhang, Y., Li, Y. & Gao, F. Selective synthesis of nickel oxide nanowires and length effect on their electrochemical properties. *Nanoscale* **2**, 920-922 (2010).

8 Xia, X. *et al.* Graphene sheet/porous NiO hybrid film for supercapacitor applications. *Chemistry–A European Journal* **17**, 10898-10905 (2011).

9 Xia, X.-h., Tu, J.-p., Wang, X.-l., Gu, C.-d. & Zhao, X.-b. Hierarchically porous NiO film grown by chemical bath deposition via a colloidal crystal template as an electrochemical pseudocapacitor material. *Journal of Materials Chemistry* **21**, 671-679 (2011).

10 Yan, X. *et al.* Rational synthesis of hierarchically porous NiO hollow spheres and their supercapacitor application. *Materials Letters* **95**, 1-4 (2013).

11 Wang, X. *et al.* High supercapacitor and adsorption behaviors of flower-like MoS 2 nanostructures. *Journal of Materials Chemistry A* **2**, 15958-15963 (2014).

12 Wei, C. *et al.* Two-dimensional β-MnO 2 nanowire network with enhanced electrochemical capacitance. *Scientific reports* **3**, 2193 (2013).

13 Luo, P. *et al.* Targeted synthesis of unique nickel sulfide (NiS, NiS2) microarchitectures and the applications for the enhanced water splitting system. *ACS applied materials & interfaces* **9**, 2500-2508 (2017).

14 Yang, L. *et al.* An efficient NiS@ N/SC hybrid oxygen evolution electrocatalyst derived from metal-organic framework. *Electrochimica Acta* **191**, 813-820 (2016).

15 Ouyang, C. *et al.* Hierarchically porous Ni3S2 nanorod array foam as highly efficient electrocatalyst for hydrogen evolution reaction and oxygen evolution reaction. *Electrochimica Acta* **174**, 297-301 (2015).

16 Zhang, D. *et al.* Ni3S2 nanowires grown on nickel foam as an efficient bifunctional electrocatalyst for water splitting with greatly practical prospects. *Nanotechnology* **29**, 245402 (2018).

17 Zhou, W. *et al.* Ni 3 S 2 nanorods/Ni foam composite electrode with low overpotential for electrocatalytic oxygen evolution. *Energy & Environmental Science* **6**, 2921-2924 (2013).

18 Zhang, J. *et al.* Interface Engineering of MoS2/Ni3S2 Heterostructures for Highly Enhanced Electrochemical Overall‐Water‐Splitting Activity. *Angewandte Chemie* **128**, 6814-6819 (2016).

19 Stern, L.-A. & Hu, X. Enhanced oxygen evolution activity by NiOx and Ni (OH) 2 nanoparticles. *Faraday discussions* **176**, 363-379 (2015).

20 Liu, Q., Gu, S. & Li, C. M. Electrodeposition of nickel–phosphorus nanoparticles film as a Janus electrocatalyst for electro-splitting of water. *Journal of Power Sources* **299**, 342-346 (2015).

21 Chen, R., Wang, H.-Y., Miao, J., Yang, H. & Liu, B. A flexible high-performance oxygen evolution electrode with three-dimensional NiCo2O4 core-shell nanowires. *Nano Energy* **11**, 333-340 (2015).

22 Xu, X., Song, F. & Hu, X. A nickel iron diselenide-derived efficient oxygen-evolution catalyst. *Nature communications* **7**, 12324 (2016).

23 Pan, Y., Chen, Y., Li, X., Liu, Y. & Liu, C. Nanostructured nickel sulfides: phase evolution, characterization and electrocatalytic properties for the hydrogen evolution reaction. *RSC Advances* **5**, 104740-104749 (2015).

24 Jiang, N. *et al.* Nickel sulfides for electrocatalytic hydrogen evolution under alkaline conditions: a case study of crystalline NiS, NiS 2, and Ni 3 S 2 nanoparticles. *Catalysis Science & Technology* **6**, 1077-1084 (2016).

25 Tang, C., Pu, Z., Liu, Q., Asiri, A. M. & Sun, X. NiS2 nanosheets array grown on carbon cloth as an efficient 3D hydrogen evolution cathode. *Electrochimica Acta* **153**, 508-514 (2015).

26 Liu, P., Li, J., Lu, Y. & Xiang, B. Facile synthesis of NiS2 nanowires and its efficient electrocatalytic performance for hydrogen evolution reaction. *International Journal of Hydrogen Energy* **43**, 72-77 (2018).

27 Fang, W., Liu, D., Lu, Q., Sun, X. & Asiri, A. M. Nickel promoted cobalt disulfide nanowire array supported on carbon cloth: an efficient and stable bifunctional electrocatalyst for full water splitting. *Electrochemistry Communications* **63**, 60-64 (2016).

28 Tang, C. *et al.* Ni3S2 nanosheets array supported on Ni foam: A novel efficient three-dimensional hydrogen-evolving electrocatalyst in both neutral and basic solutions. *International Journal of Hydrogen Energy* **40**, 4727-4732 (2015).

29 Chen, R. *et al.* Porous nickel disulfide/reduced graphene oxide nanohybrids with improved electrocatalytic performance for hydrogen evolution. *Catalysis Communications* **85**, 26-29 (2016).

30 Zhu, W. *et al.* Nickel sulfide microsphere film on Ni foam as an efficient bifunctional electrocatalyst for overall water splitting. *Chemical Communications* **52**, 1486-1489 (2016).
